# Extended Endocrine Therapy Following 5 Years of Adjuvant Luteinizing Hormone-Releasing Hormone Agonist in Premenopausal Patients With Node-Positive, Hormone Receptor–Positive Breast Cancer: A Cohort Study

**DOI:** 10.1200/JCO-25-01660

**Published:** 2026-01-15

**Authors:** Carmine Valenza, Yue Zheng, Monica Milano, Dario Trapani, Elisa Giordano, Lorenzo Guidi, Pier Paolo Maria Berton Giachetti, Laura Boldrini, Grazia Castellano, Jalissa Katrini, Bianca Malagutti, Gabriele Antonarelli, Fabio Conforti, Eleonora Pagan, Vincenzo Bagnardi, Gregory J. Kirkner, Claudia Sangalli, Kate E. Dibble, Marco Colleoni, Meredith M. Regan, Elisabetta Munzone, Giuseppe Curigliano, Ann H. Partridge

**Affiliations:** ^1^ Harvard Chan School of Public Health, Harvard University, Boston, MA; ^2^ Division of New Drugs and Early Drug Development for Innovative Therapies, European Institute of Oncology, IRCCS, Milan, Italy; ^3^ Department of Oncology and Hemato-Oncology, University of Milan, Milan, Italy; ^4^ Department of Medical Oncology, Dana-Farber Cancer Institute, Boston, MA; ^5^ Breast Oncology Program, Dana-Farber Brigham Cancer Center, Boston, MA; ^6^ Department of Data Science, Dana-Farber Cancer Institute, Boston, MA; ^7^ Division of Medical Senology, European Institute of Oncology, IRCCS, Milan, Italy; ^8^ Division of Medical Oncology, Humanitas Gavazzeni, Bergamo, Italy; ^9^ Department of Statistics and Quantitative Methods, University of Milano-Bicocca, Milan, Italy; ^10^ Clinical Trial Office, European Institute of Oncology, IRCCS, Milan, Italy; ^11^ Harvard Medical School, Boston, MA

## Abstract

**PURPOSE:**

To evaluate the clinical benefit of extended endocrine therapy (eET) after 5 years of adjuvant treatment with luteinizing hormone-releasing hormone agonists (LHRHa) in premenopausal women with node-positive, hormone receptor–positive early breast cancer (eBC).

**METHODS:**

We conducted a cohort study analysis on two prospectively collected data sets (the Young Women's Breast Cancer Study and IEO Breast Cancer Cohort). Eligible patients were diagnosed with eBC at age ≤40 years (between 2005 and 2016), had node-positive, hormone receptor–positive disease, and remained premenopausal after 5 years of adjuvant LHRHa with no evidence of recurrence. The primary end point was invasive breast cancer–free survival (IBCFS), calculated from the sixth year after the initiation of adjuvant endocrine therapy (ET; study baseline), and adjusted through the propensity score (PS) weighting analysis.

**RESULTS:**

A total of 501 patients were included in the analysis: 287 received eET for a median duration of 3.7 years (IQR, 2.3-5.0), including 48% tamoxifen monotherapy and 52% LHRHa plus tamoxifen or aromatase inhibitor. After a median follow-up of 7.3 years from the study baseline, the PS weighted IBCFS rates at 5 years were 85% in the eET group and 78% in the non-eET group (hazard ratio [HR], 0.63 [95% CI, 0.44 to 0.89]; *P* = .0135). The PS weighted distant recurrence-free survival rates at 5 years were 91% and 83% in the eET and non-eET group, respectively (cause-specific HR, 0.49 [95% CI, 0.31 to 0.79]). In both groups, bone fractures and major cardiovascular events were reported in 1% of patients.

**CONCLUSION:**

In this cohort study analysis, extending ET in premenopausal patients with node-positive eBC after 5 years of LHRHa treatment was associated with a clinically meaningful reduction in both invasive and distant breast cancer recurrences.

## INTRODUCTION

Breast cancer (BC) is the leading cause of cancer-related morbidity, disability, and mortality among young women, aged 40 years or younger at diagnosis.^1^ In this population, which has been steadily increasing worldwide over the past 2 decades, BC is more often diagnosed at an advanced stage and is more frequently associated with aggressive tumor subtypes than in older women, contributing to a poorer overall prognosis.^2^ Younger age at diagnosis has also been identified as an independent risk factor for recurrence, particularly among patients with hormone receptor–positive early BC (eBC).^3,4^

**CONTEXT**

**Key Objective**
To evaluate the clinical benefit of extended endocrine therapy (eET) after 5 years of adjuvant treatment with luteinizing hormone-releasing hormone agonists (LHRHa) in premenopausal women with node-positive, hormone receptor–positive early breast cancer.
**Knowledge Generated**
In this cohort study on 501 patients who remained premenopausal after 5 years of LHRHa treatment, the extension of endocrine therapy either with tamoxifen alone or by continuing previous LHRHa was associated with reduced invasive and distant breast cancer (BC) recurrences. Bone fractures and major cardiovascular events were reported in fewer than 1% of patients during the study period, regardless of the initiation of eET.
**Relevance *(K.D. Miller)***
These data fill a critical gap in our knowledge of the optimal treatment of premenopausal women with hormone-sensitive BC.*
*Relevance section written by *JCO* Senior Deputy Editor Kathy D. Miller, MD.

Adjuvant endocrine therapy (ET) with 5 years of tamoxifen demonstrated to improve overall survival (OS) in premenopausal women with hormone receptor–positive eBC, compared with no ET.^5^ The SOFT trial subsequently showed that the addition of ovarian function suppression (OFS) with a luteinizing hormone-releasing hormone agonist (LHRHa) to tamoxifen or an aromatase inhibitor (AI) for 5 years further decreases BC mortality compared with tamoxifen alone.^6^

However, among premenopausal patients with high-risk, hormone receptor–positive eBC treated with OFS plus AI, the risk of distant recurrence approaches 25% at 15 years, highlighting the urgent need to reduce late recurrences by optimizing ET in this subgroup.^7,8^

In premenopausal and postmenopausal patients, the aTTom and ATLAS trials demonstrated that extending adjuvant tamoxifen to 10 years further decreases BC-specific mortality.^9,10^ However, no evidence currently supports the extension of ET in patients who remain premenopausal after completing 5 years of adjuvant therapy with a LHRHa.^11^ In clinical practice, extended ET (eET) is recommended to over a half of these patients, according to the risk of recurrence and their preferences and consists of either switching to tamoxifen monotherapy or continuing LHRHa (plus tamoxifen or AI).^12^ At the 17th St Gallen International Breast Cancer Conference (2021), 87% of panelists supported consideration of eET for premenopausal women with node-positive, hormone receptor–positive eBC, based on an expert opinion; 42% favored continuation of OFS while 45% recommended tamoxifen monotherapy.^13^

This study aims to evaluate the benefit of eET in patients with node-positive, hormone receptor–positive eBC who remained premenopausal after completing 5 years of adjuvant ET with LHRHa treatment.

## METHODS

### Study Design

We conducted an international, multicenter cohort study analysis using two prospectively-maintained data sets: the Young Women's Breast Cancer Study (YWS) and the European Institute of Oncology (IEO) Breast Cancer cohort. The YWS is a multicenter, prospective cohort of women diagnosed with BC at ≤40 years of age, enrolled a median of 4 months after diagnosis between 2006 and 2016 across 13 sites in the United States and Canada.^12^ The IEO Breast Cancer cohort is a single-center, prospectively maintained data set of patients who underwent surgery for BC at the IEO, an academic cancer center in Milan, Italy, beginning in 1994. Follow-up in both cohorts is ongoing.

For the YWS, a combination of medical record review and patient surveys is used for patient and disease characteristics, treatments, as well as outcomes during the follow-up. Medical record review is used for the ascertainment of these data in the IEO cohort.

All participants provided written informed consent to enter the cohorts. The current joint analysis had been approved by IEO institutional review board (UID: 4672) and was conducted in accordance with the principles of the Declaration of Helsinki and Good Clinical Practice guidelines.

### Participants

We included women diagnosed with eBC at age ≤40 years between 2005 and 2016, who underwent breast surgery for tumors of ductal, lobular, or ductolobular histology. Eligible participants had node-positive, nonmetastatic disease (defined as pT-any, pN1-3, cM0, according to the AJCC TNM 8th edition), and were classified as hormone receptor–positive/HER2-any subtype (estrogen and/or progesterone receptor ≥1% by immunohistochemistry).

Patients were required to have completed 5 years of adjuvant ET with LHRHa, with no evidence of distant or locoregional recurrence (either invasive or noninvasive) at that time (as per clinical practice, no routine whole-body staging was performed after the first 5 years of ET unless clinically indicated).

They also needed to remain premenopausal following the completion of the first 5 years of adjuvant ET, based on at least one of the following criteria: (1) age <45 years, (2) plasma estradiol levels within the premenopausal range within 6 months after discontinuation of the LHRH-agonist (patients could be on or off ET, such as tamoxifen, at the time of this blood test), and (3) recovery of menstruation after discontinuation of the LHRHa. Patients were excluded if they had undergone bilateral oophorectomy or radiotherapy ovarian ablation or had a history of invasive BC before the index diagnosis.

### Exposure and Outcomes

The exposure was the initiation of eET (with tamoxifen monotherapy, LHRHa plus tamoxifen, or LHRHa plus AI), irrespective of the treatment duration of eET, measured at study baseline (defined as the first day of the 6th year after the initiation of adjuvant ET).

The main end point was invasive breast cancer–free survival (IBCFS), defined as the time from the study baseline to the occurrence of ipsilateral invasive BC recurrence, contralateral invasive BC recurrence, local-regional invasive BC recurrence, distant BC recurrence, or death, whichever occurred first.^14^

Other end points included the following: distant recurrence-free survival (DRFS), measured as the time from the study baseline to distant BC recurrence as the first BC event, or death, whichever occurs first; patients experiencing at least one bone fracture during the study period; and patients experiencing at least one major cardiovascular event during the study period, including unstable angina, nonfatal myocardial infarction, nonfatal stroke, cardio-cerebrovascular death, myocardial infarction, or stroke.^15^

As per standard clinical practice, the standard clinical follow-up interval for patients in this cohort was at least every 12 months (with mammography and/or ultrasound, physical examination). All patients with locoregional recurrences underwent whole body staging and tissue biopsy; otherwise, the investigation of distant events was based on signs and symptoms. Data on bone fractures and major cardiovascular events were systematically reported during follow-up visits and were collected through comprehensive review of medical records.

### Statistical Analysis

Descriptive statistics were used to analyze patients' characteristics. Continuous variables were expressed as the median and IQR. Categorical variables are expressed as numbers and proportions (%).

IBCFS and DRFS distributions were estimated using the adjusted Kaplan-Meier method among patients with and without the exposure, weighted through the propensity score (PS) weighting analysis (with stabilized weights)^16^ for the following a priori defined covariates (scientific approach): cohort (YWS *v* IEO), age at diagnosis, tumor histology (ductal *v* lobular or ductolobular), tumor dimension (pT1 *v* pT2 *v* pT3/4), nodal status (pN1 *v* pN2/pN3), disease subtype (luminal A-like *v* luminal B-like disease), type of adjuvant ET received during the first 5 years (LHRHa plus AI *v* LHRHa plus tamoxifen or LHRHa alone), and receipt of adjuvant chemotherapy (yes *v* no). Luminal B-like disease was defined as HER2-positive and/or grade 3 disease, according to the 12th St Gallen International Breast Cancer Conference (2011).^17^

To account for the potential effect on distant recurrences of ET switch or reinitiation after ipsilateral invasive breast tumor recurrence, contralateral invasive breast tumor recurrence, and locoregional invasive recurrence, DRFS was analyzed with the cause-specific hazard ratio (HR) approach.

Median follow-up was calculated from the study baseline by the reverse Kaplan-Meier method, censoring four patients who underwent oophorectomy after the study baseline. Median duration of eET was summarized as observed from the study baseline to the end of reported taking eET or the last follow-up (if eET ongoing) among patients in the eET group.

We fit a PS weighted Cox regression model (with the same stabilized PS weights as the PS model described above) to assess the effect of eET on IBCFS and on DRFS across the following subgroups: histology (ductal, and lobular or ductolobular), tumor dimension (pT1, pT2, and pT3/4), nodal status (pN1 and pN2/pN3), disease subtype (luminal A-like and luminal B-like disease), HER2 status (HER2-positive and HER2-negative), and type of adjuvant ET received during the first 5 years (LHRHa plus AI and LHRHa plus tamoxifen or LHRHa alone). Interaction tests were performed, and 95% CI for the PS weighted HRs were estimated using robust SE.^18^

Receipt of adjuvant chemotherapy was not considered as a potential effect modifier on the basis of the evidence that chemotherapy has not a relative impact on IBCFS events beyond the fifth year.^19^

To account for a potential difference in median follow-up between the eET group and the non-eET group, because of the increasing likelihood in prescribing eET in the last years, we performed a sensitivity analysis of IBCFS excluding the subgroup of patients whose study baseline was after 2019.

All statistical tests were two-sided, with *P* < .05 considered statistically significant. No adjustment for multiplicity was performed. Analyses were conducted using SAS (version 9.4, SAS Institute, Cary, NC).

## RESULTS

**FIG 1.**
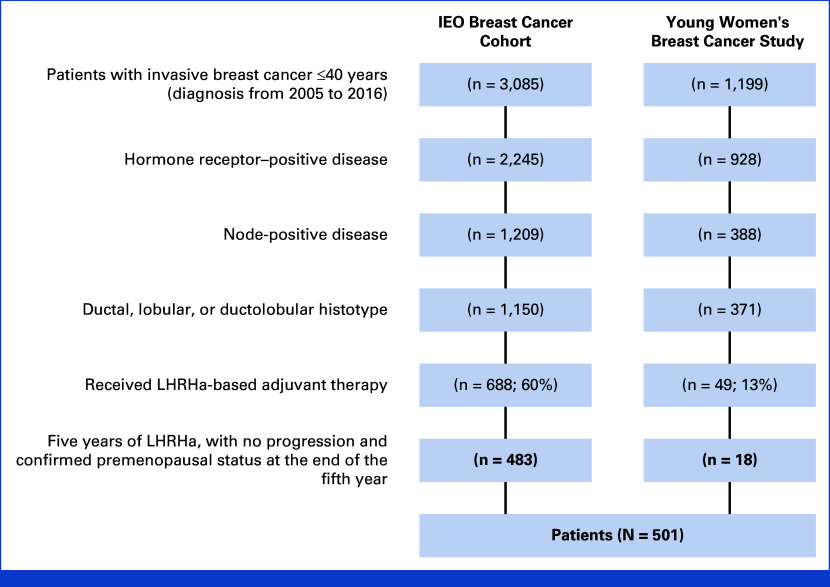
Flow diagram. LHRHa, luteinizing hormone-releasing hormone agonist.

**FIG 2.**
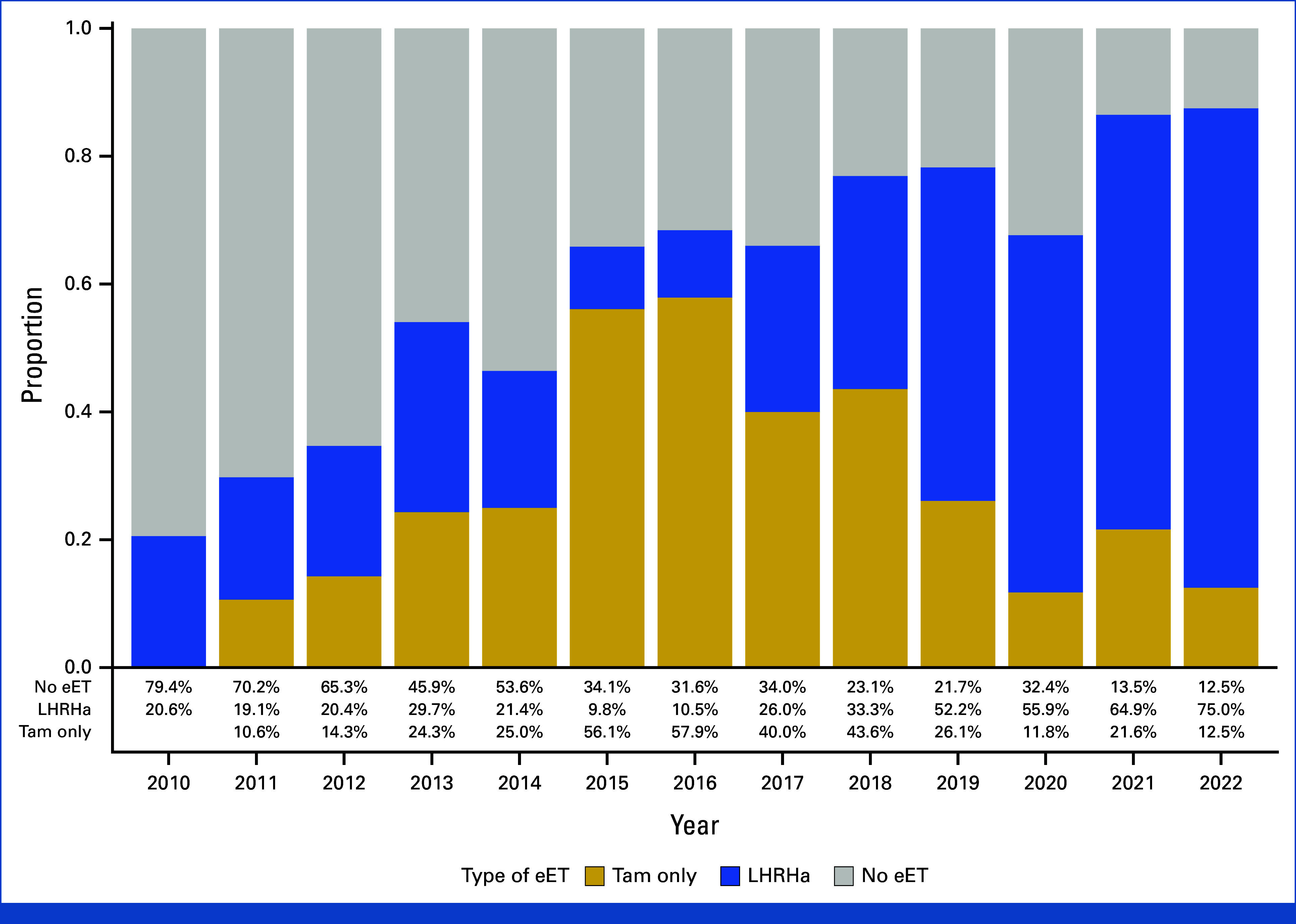
Proportions of patients who did or did not receive eET, according to year of study baseline (2010-2022), among those with node-positive eBC who remained premenopausal after completing 5 years of LHRH agonist-based adjuvant ET. eBC, early breast cancer; ET, endocrine therapy; eET, extended endocrine therapy; LHRHa, luteinizing hormone-releasing hormone agonist; Tam, tamoxifen.

Overall, 3,085 patients from the IEO and 1,199 from the YWS were screened, and 501 eligible patients were included (Fig 1). Among them, 287 (57%) received eET, while 214 (43%) underwent follow-up after 5 years of ET including LHRHa. The proportion of prescription of eET increased from 21% in 2010 to 88% in 2022 (Fig 2).

Disease, demographic, and treatment data are presented in Table 1 (the PS weighted analysis is reported in the Data Supplement, Tables S1 and S2). Compared with the non-eET group, more patients in the eET group had pT3 or pT4 disease (14% *v* 7%), pN2 or pN3 nodal stage (36% *v* 26%), and grade 3 tumor (49% *v* 42%). Half of the patients in both groups had a luminal B-like disease.

**TABLE 1.** Characteristics of Patients With Node-Positive, Hormone Receptor–Positive Early Breast Cancer Who Completed 5 Years of LHRH Agonist-Based Adjuvant Endocrine Therapy and Were Assessed for Extended Endocrine Therapy Use

Characteristic	Extended Endocrine Therapy (n = 287)	No Extended Endocrine Therapy (n = 214)
Patient characteristics		
Age at diagnosis, years, median (IQR)	37 (35-39)	37 (33-39)
BMI at diagnosis, median (IQR)	22 (20-24)	21 (20-23)
Germline *BRCA* status, No. (%)		
Mutated	11 (4)	8 (4)
Wild type	126 (44)	57 (27)
Not assessed	150 (52)	149 (69)
Cohort, No. (%)		
IEO	273 (95)	210 (98)
YWS	14 (5)	4 (2)
Tumor characteristics, No. (%)		
Histology		
Ductal	262 (91)	203 (95)
Lobular	13 (5)	6 (3)
Ductolobular	12 (4)	5 (2)
Tumor stage		
pT1	107 (37)	88 (41)
pT2	139 (49)	112 (52)
pT3/4	41 (14)	14 (7)
Nodal stage		
pN1	184 (64)	158 (74)
pN2	63 (22)	35 (16)
pN3	40 (14)	21 (10)
Grade		
Grade 1	8 (3)	8 (4)
Grade 2	137 (48)	115 (54)
Grade 3	141 (49)	90 (42)
NA	1 (<1)	1 (<1)
Multifocal disease	88 (31)	60 (28)
HER2-positive	54 (19)	46 (21)
Surrogate biologic subtype		
Luminal A-like	134 (47)	105 (49)
Luminal B-like (HER2+ or grade 3)	153 (53)	109 (51)
Previous anticancer treatments, No. (%)		
ET during the first 5 years		
LHRH-agonist plus tamoxifen	188 (65)	165 (77)
LHRH-agonist plus AI	97 (34)	46 (22)
LHRH-agonist only	2 (<1)	3 (1)
Previous chemotherapy		
Anthracyclines and taxanes	112 (39)	51 (24)
Anthracyclines and CMF	3 (1)	5 (2)
Anthracyclines	84 (29)	83 (39)
CMF	2 (<1)	2 (<1)
Taxanes	4 (1)	1 (<1)
Other	17 (6)	8 (4)
No chemotherapy	65 (23)	64 (30)
Previous anti-HER2 agents	53/54 (98)	42/46 (91)
Previous radiotherapy		
Yes	184 (64)	133 (62)
No	76 (26)	57 (27)
IORT	27 (9)	24 (11)
Bone health agents during the first 5 years of ET	19 (7)	5 (2)

Abbreviations: AI, aromatase inhibitor; CMF, cyclophosphamide, methotrexate, and fluorouracil; ET, endocrine therapy; IEO, European Institute of Oncology; IORT, intraoperative radiotherapy; LHRH, luteinizing hormone-releasing hormone; NA, not available; YWS, Young Women's Breast Cancer Study.

During the first 5 years of adjuvant ET, 188 (65%) patients from the eET group and 165 (77%) from the non-eET received LHRHa plus tamoxifen, and 97 (34%) and 46 (22%) received LHRHa plus AI, respectively. In the eET group, 222 (77%) patients received chemotherapy compared with 150 (70%) patients in the non-eET group; anthracycline-based chemotherapy was the most common prescribed regimen. More than 90% of patients with HER2-positive disease received adjuvant trastuzumab, in both groups.

In the eET group (n = 287), the median duration of eET was 3.7 years (IQR, 2.3-5.0): 137 of 287 (48%) received eET with tamoxifen monotherapy for a median duration of 4.0 years (IQR, 2.0-5.0) and 150 of 287 (52%) received eET with LHRHa plus tamoxifen or an AI for a median duration of 3.6 years (IQR, 2.3-5.0). Furthermore, in this eET group, 19 of 287 (7%) received a concomitant bone health agent for osteoporosis (bisphosphonate or denosumab).

**FIG 3.**
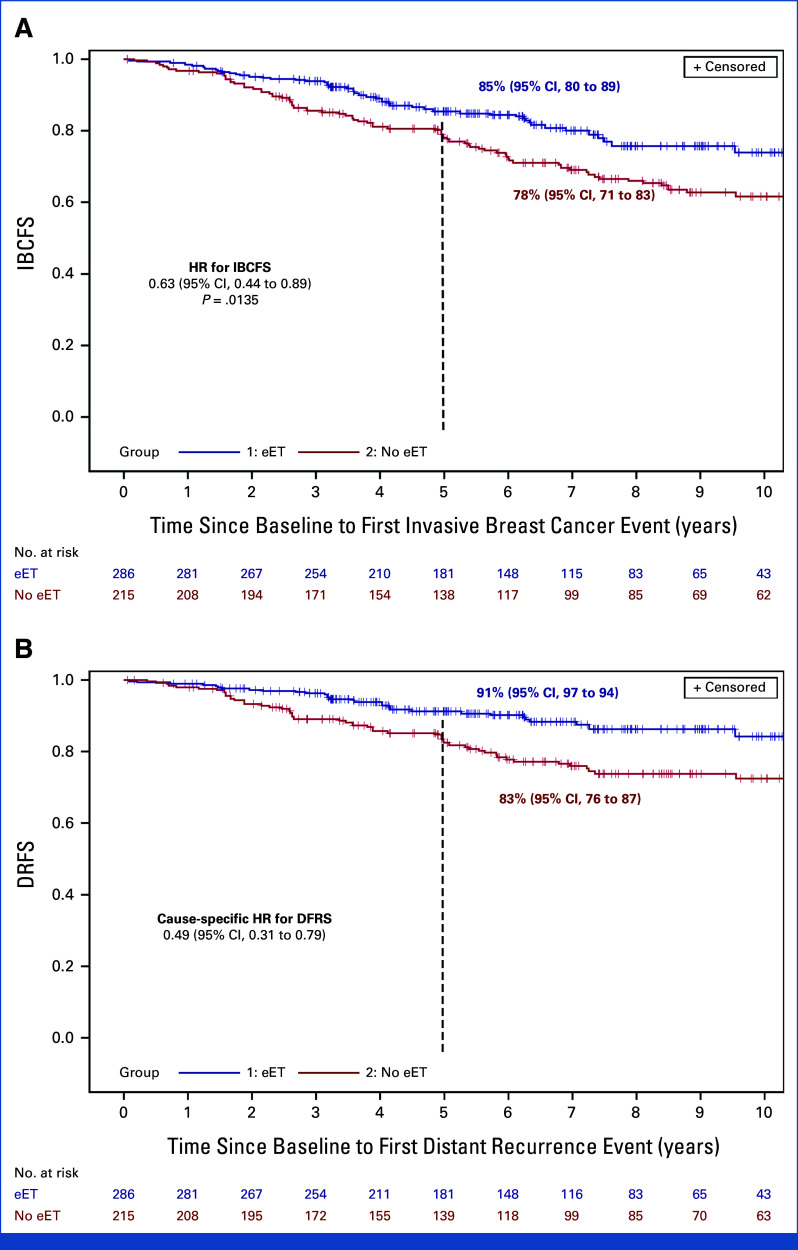
Adjusted Kaplan-Meier curve of (A) IBCFS and (B) DRFS by receipt of eET after PS-weighted analysis, in patients with node-positive, hormone receptor–positive eBC who completed 5 years of LHRH agonist-based adjuvant ET. DRFS, distant recurrence-free survival; eBC, early breast cancer; ET, endocrine therapy; eET, extended endocrine therapy; HR, hazard ratio; IBCFS, invasive breast cancer-free survival; PS, propensity score.

**FIG 4.**
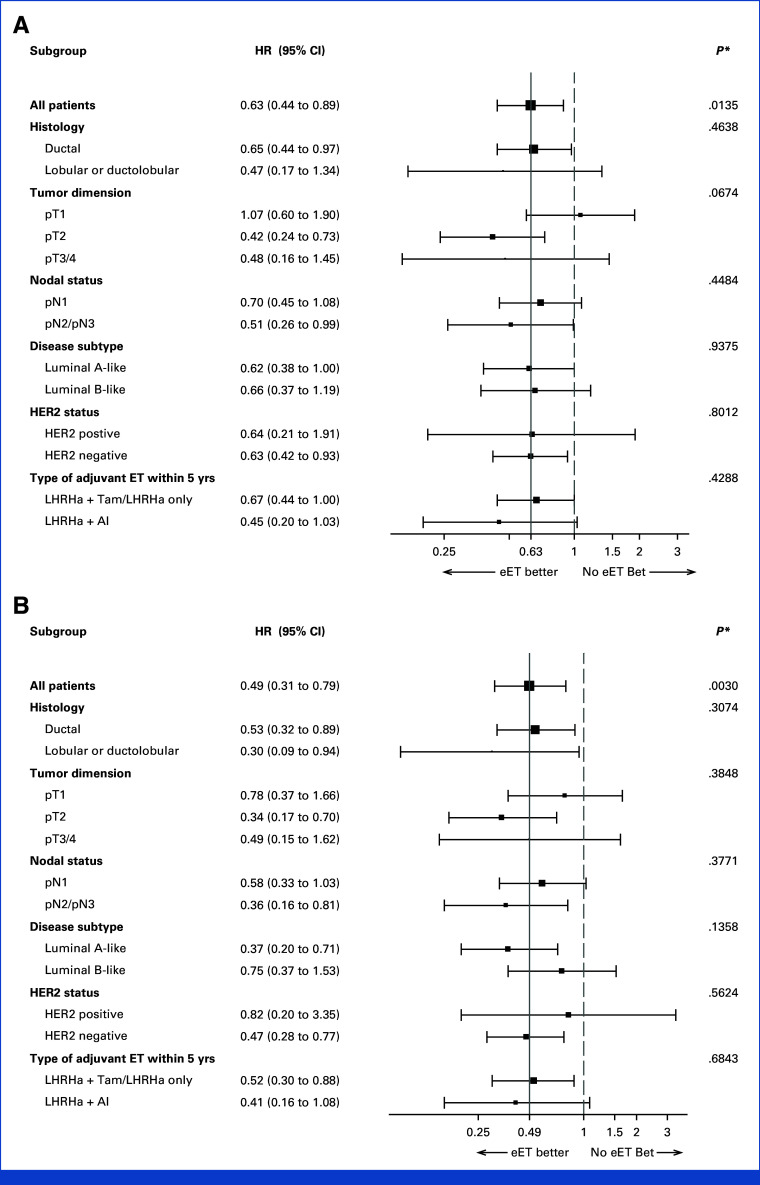
Subgroup analyses for receipt of eET on (A) IBCFS and (B) DRFS after PS-weighted analysis, in patients with node-positive, hormone receptor–positive eBC who completed 5 years of LHRH agonist-based adjuvant endocrine therapy. AI, aromatase inhibitor; DRFS, distant recurrence-free survival; eBC, early breast cancer; ET, endocrine therapy; eET, extended endocrine therapy; HR, hazard ratio; IBCFS, invasive breast cancer–free survival; LHRHa, LHRH agonist; PS, propensity score; Tam, tamoxifen. *P value is the test of interaction between treatment and each subgroup unadjusted for multiplicity.

After a median follow-up of 7.3 years (IQR, 4.8-10.3), 53 and 74 IBCFS events occurred in the eET and non-eET groups, respectively (Table 2). The PS weighted HR for IBCFS comparing the eET to the non-eET group was 0.63 ([95% CI, 0.44 to 0.89]; *P* = .0135; Fig 3A). After 5 years from study baseline, the PS weighted IBCFS rates were 85% (95% CI, 80 to 89) and 78% (95% CI, 71 to 83), respectively. This benefit appeared consistent across the explored subgroups, with the possible exception of patients with pT1 disease (Fig 4A).

**TABLE 2.** Summary of First Events in the Analysis of Invasive Breast Cancer-Free Survival and of Safety Events in Patients With Node-Positive, Hormone Receptor–Positive Early Breast Cancer Who Completed 5 Years of LHRH Agonist-Based Adjuvant Endocrine Therapy and Were Assessed for Extended Endocrine Therapy Use

Events	Extended Endocrine Therapy (n = 287), No. (%)	No Extended Endocrine Therapy (n = 214), No. (%)
IBCFS (first event)	53 (19)	74 (34)
Ipsilateral invasive breast tumor recurrence	5 (2)	11 (5)
Contralateral invasive breast tumor recurrence	14 (5)	8 (4)
Locoregional invasive recurrence	6 (2)	8 (4)
Distant recurrence	28 (10)	44 (21)
Death	0 (0)	1 (<1)
Safety events		
Bone fractures	4 (1)	2 (1)
Major cardiovascular events	3 (1)	3 (1)

Abbreviations: IBCFS, invasive breast cancer-free survival; LHRH, luteinizing hormone-releasing hormone.

Twenty-eight and 44 DRFS events occurred in the eET and non-eET groups as first event, respectively. The PS-weighted cause-specific HR for DRFS comparing the eET with the non-eET group was 0.49 ([95% CI, 0.31 to 0.79]; Fig 3B). After 5 years from study baseline, the PS-weighted DRFS rates were 91% (95% CI, 87 to 94) and 83% (95% CI, 76 to 87) respectively. This benefit appeared consistent across all subgroups (Fig 4B).

During the study period, bone fractures were reported in four patients (1%) in the eET group and in two (1%) in the non-eET group. Major cardiovascular events were reported in three patients (1%) in the eET group and in three (1%) in the non-eET group. Only one death without recurrence occurred in the non-eET group.

The increasing rate of eET prescriptions from 2010 to 2022 resulted in a clinically significant difference in median follow-up duration comparing the eET group (6.4 years; IQR, 4.4-9.1) and the non-eET group (8.4 years; IQR, 5.4-11.3). Therefore, we conducted a sensitivity analysis of IBCFS, excluding 87 patients whose study baseline was after 2019, which showed a consistent PS weighted HR for IBCFS comparing the eET with the non-eET group of 0.64 ([95% CI, 0.44 to 0.93]; Data Supplement, Fig S1).

## DISCUSSION

In this study, we found that extending ET beyond the fifth year in premenopausal patients with node-positive hormone receptor–positive eBC was associated with a clinically meaningful reduction in the risk of an invasive BC recurrence and improved DRFS. The only prior evidence regarding eET efficacy in the premenopausal setting comes from the subgroup analysis of the ATLAS trial (n = 630/6,846, 9.2%), which showed a recurrence rate ratio of 0.81 comparing eET with tamoxifen with no eET (after 5 years of tamoxifen monotherapy).^10^

In this study, the benefits in IBCFS and DRFS associated with eET appear to be consistent across all subgroups of premenopausal women with a history of node-positive disease. Speculatively, tumors with higher stage (ie, pT2 or pT3/4 compared with pT1, and pN2/3 compared with pN1) and lobular cancers may derive a greater benefit from the extension of ET. However, larger studies are needed to confirm these observations.

No differences in bone fractures, major cardiovascular events, or deaths without recurrence (because of potential long-term detrimental effects of ovarian suppression) were reported between the two groups during the study period, consistent with the SOFT trial and a patient-level meta-analysis on 14,999 women.^6,20^ However, longer follow-up of the current cohort is required to draw definitive conclusions regarding adverse events with eET. A cohort study on premenopausal women without eBC found that oophorectomy before age 46 years was associated with an increased risk of chronic conditions, including cardiac arrhythmias, coronary artery disease, and osteoporosis.^21^ Decisions regarding eET should consider not only the remaining risk of disease recurrence and these serious adverse events but also persistent side effects which may adversely affect quality of life, not reported in our study (ie, vasomotor symptoms, psychosocial and sexual concerns). Accordingly, we believe that the shared decision-making process should also take into account self-reported toxicity and overall distress to inform the prescription of eET.

We observed that the proportion of patients who received eET progressively increased from 21% in 2010 to 88% in 2022, because of the growing evidence supporting the benefit of eET in postmenopausal patients with hormone receptor–positive, node-positive eBC, which has been extrapolated into the premenopausal setting. Additionally, the demonstration of an OS benefit from intensifying ET with LHRHa in the SOFT trial in 2018 further supported this trend.^20,22^

Previously, between 2009 and 2013, a phase II study evaluating 2 years of LHRHa-based eET in premenopausal patients after adjuvant tamoxifen was closed because of poor accrual.^23^ The receipt of eET grew to 60% among patients who were candidates for eET in the YWS between 2011 and 2021, primarily with tamoxifen after tamoxifen.^12^ The acceptability of eET with LHRHa after prior LHRHa was first assessed in 2023, through a survey on 615 young patients with eBC receiving adjuvant LHRHa: 64% of them reported that they would consider extending LHRHa therapy to increase the probability of disease-free survival.^24^

This study has several strengths and limitations. It assessed the extension of ET with a median follow-up of 7 years from eET initiation, including patients from two prospective cohorts. Given the current prescription rate of eET in premenopausal patients with node-positive eBC (>80%) and the benefits observed in the postmenopausal setting, it would no longer be feasible or ethical to address the value of eET through a randomized clinical trial.

The premenopausal status at the study baseline was predefined by clinical and/or laboratory criteria, reflecting the current clinical practice and the ongoing clinical trials, recognizing that these criteria do not guarantee that a woman was actually premenopausal at the time.^25-27^

The contribution of the two cohorts was unbalanced (96% from IEO *v* 4% from YWS) because of the different proportion of prescription of LHRHa for the first 5 years (60% *v* 13%, respectively), likely reflecting the regional oncology practice at the times that the cohorts were assembled.

The eET and non-eET groups were balanced by PS analysis, although this does not ensure the same balance as a proper random assignment.^28^ Owing to the small number of events and the immature follow-up for OS, we did not assess OS, nor did we evaluate the optimal duration of eET or compare different eET strategies (ie, tamoxifen monotherapy *v* continuation of LHRHa). Larger prospective randomized clinical trials are required to address these important questions.

Despite the difference in median follow-up between the eET and the non-eET group, because of the increasing rate of eET prescription from 2010 to 2022, the sensitivity analysis confirmed that the magnitude of clinical benefit associated with the extension of ET was consistent with our primary analysis.

None of the patients received adjuvant CDK4/6 inhibitors nor olaparib, which may have led to a dilution of the effect of eET, because of the higher efficacy of these agents compared with ET alone, and their potential carryover effects.^29,30^

Adherence to eET was not assessed in this study, despite evidence indicating its impact on disease-free survival, particularly in young women with BC.^4,31^ However, we included a population that was expected to be adherent as all participants had received LHRH agonists for 5 years; the lack of adherence adjustment would likely result only in an underestimation of the eET benefit. Adherence to mammographic surveillance during the follow-up was also not collected.

Future challenges include the incorporation into the adjuvant setting of novel endocrine agents, such as oral selective estrogen receptor degraders, currently under evaluation in several phase III trials (also as extended therapy).^32^ These agents demonstrated superiority over AIs in the metastatic setting and may address the potentially limited antitumoral activity of LHRHa in case of incomplete OFS, described in 20% of premenopausal patients.^33,34^

The presence of circulating tumor cells or DNA was shown to be associated with metastatic recurrence in patients with high-risk, hormone receptor–positive eBC more than 5 years after diagnosis.^35,36^ If adequately validated in prospective clinical trials, these biomarkers could optimize clinical decisions regarding eET.

In conclusion, in this cohort study analysis, extending ET in patients with node-positive, hormone receptor–positive eBC who remained premenopausal after 5 years of LHRHa treatment, led to a clinically meaningful improvement in IBCFS and DRFS.

## Data Availability

A data sharing statement provided by the authors is available with this article at DOI https://doi.org/10.1200/JCO-25-01660. Data will be available for sharing with researchers who provide a methodologically sound proposal after proper revision of the data transfer agreement of each participating center and if ultimately allowed by the local ethics committee. The types of analyses allowed will be those able to achieve the aims of the approved proposal. Proposals should be directed to: ann_partridge@dfci.harvard.edu.
