# Supplementary material for: Extended Endocrine Therapy Following 5 Years of Adjuvant Luteinizing Hormone-Releasing Hormone Agonist in Premenopausal Patients With Node-Positive, Hormone Receptor–Positive Breast Cancer: A Cohort Study
Source: J Clin Oncol. 2026 Jan 15;44(6):486–96. doi: 10.1200/JCO-25-01660 (PMC12904225; doi:10.1200/JCO-25-01660)
Supplement: Supplementary file 1 [file jco-44-486-s001.pdf]

ASCO Journals Data Sharing Statement Questionnaire

**1. Manuscript Title**	Extended Endocrine Therapy Following Five Years of Adjuvant LHRH-agonist in Premenopausal Patients with Node-Positive, Hormone Recptor-Positive Breast Cancer: A Cohort Study
**2. First Author Last Name**	Valenza
**3. Does your manuscript use ONLY data from a publicly available database (eg, SEER, Medicare)?** If "Yes," please specify the database; no additional information is needed. If "No," please complete questions 4-17.	No
**4. Will the data collected for your study be made available to others?** (If "No," enter context for your decision)	Yes
**5. List which data are available**	Baseline clinical data and outcomes
**6. Additional information about the data** (Enter "None," if applicable)	None
**7. List how or where the data can be obtained** (e.g., e-mail address, URL, or other repository)	Proposals should be directed to: ann_partridge@dfci.harvard.edu
**8. List beginning and end dates that data will be available** (e.g., MM/DD/YYYY to MM/DD/YYYY)	12/1/2025 - 12/1/2026
**9. List any supporting documents** (Enter "None," if applicable)	None
**10. Enter additional information about supporting documents** (Enter "None," if applicable)	None

11. **How or where can supporting documents be obtained?** (e.g., e-mail address, URL, or other repository)	None
12. **List beginning and end dates that supporting documents will be available** (e.g., MM/DD/YYYY to MM/DD/YYYY)	None
13. **Indicate to whom data will be available** (Enter "U/K" for unknown; "N/A" for not applicable)	Data will be available for sharing with researchers who provide a methodologically sound proposal after proper revision of the data transfer agreement of each participating center and if ultimately allowed by the local ethics committee.
14. **Indicate for what type of analysis or purpose** (Enter "U/K" for unknown)	The types of analyses allowed will be those able to achieve the aims of the approved proposal.
15. **Indicate by what mechanism** (Enter "U/K" for unknown)	U/K
16. **Enter any other restrictions** (Enter "None," if applicable)	None
17. **Enter any additional information** (Enter "None," if applicable)	None

(Optional) If you have included or would like to include a Data Sharing Statement in addition to the above, please provide on the following page.

Data Sharing Statement

Data will be available for sharing with researchers who provide a methodologically sound proposal after proper revision of the data transfer agreement of each participating center and if ultimately allowed by the local ethics committee. The types of analyses allowed will be those able to achieve the aims of the approved proposal.
Proposals should be directed to: ann_partridge@dfci.harvard.edu
