# Supplementary material for: Extended Endocrine Therapy Following 5 Years of Adjuvant Luteinizing Hormone-Releasing Hormone Agonist in Premenopausal Patients With Node-Positive, Hormone Receptor–Positive Breast Cancer: A Cohort Study
Source: J Clin Oncol. 2026 Jan 15;44(6):486–96. doi: 10.1200/JCO-25-01660 (PMC12904225; doi:10.1200/JCO-25-01660)
Supplement: Supplementary file 2 [file jco-44-486-s002.pdf]

Supplementary Table 1. Characteristics of patients with node-positive, hormone receptor-positive early breast cancer who completed 5 years of LHRH agonist-based adjuvant endocrine therapy and were assessed for extended endocrine therapy use, after propensity score weighting*.

**Characteristic**	**Extended endocrine therapy**	**No extended endocrine therapy**
**Patient characteristics**		
Age at diagnosis, median (IQR)*	37 (34-39)	37 (34-39)
Body Mass Index at diagnosis, median (IQR)	22 (20-24)	21 (20-23)
Germline *BRCA* status		
Mutated, (%)	4	4
Wild-type, (%)	43	27
Not assessed, (%)	53	68
Cohort*		
IEO, (%)	96	96
YWS, (%)	4	4
**Tumor characteristics**		
Histology*		
Ductal, (%)	93	92
Lobular, (%)	3	4
Ductolobular, (%)	4	4
Tumor stage*		
pT1, (%)	39	39
pT2, (%)	49	49
pT3/4, (%)	11	12
Nodal stage*		
pN1, (%)	68	68
pN2, (%)	20	19
pN3, (%)	12	12
Grade		
Grade 1, (%)	3	3
Grade 2, (%)	49	53
Grade 3, (%)	48	43
NA, (%)	<1	<1
Multifocal disease, (%)	30	29
HER2-positive, (%)	19	21
Surrogate biological subtype*		
Luminal A-like, (%)	48	49
Luminal B-like (HER2+ or Grade 3), (%)	52	51
**Previous anticancer treatments**		
Endocrine therapy during the first 5 years*		
LHRH-agonist plus tamoxifen, (%)	71	69
LHRH-agonist plus AI, (%)	29	29
LHRH-agonist only, (%)	<1	1
Previous chemotherapy*		
Anthracyclines and taxanes, (%)	37	27
Anthracyclines and CMF, (%)	1	3
Anthracyclines, (%)	30	39

CMF, (%)	<1	1
Taxanes, (%)	1	<1
Other, (%)	5	4
No chemotherapy, (%)	26	26
Previous anti-HER2 agents, (%)	98	93
Previous radiotherapy		
Yes, (%)	63	63
No, (%)	27	27
IORT, (%)	10	11
Bone health agents during the first five years of ET, (%)	6	3

* The propensity score was calculated based on variables with asterisk, i.e.: cohort, age, histology, tumor stage. nodal stage, surrogate biological subtype, type of endocrine therapy therapy during the first 5 years, receipt of chemotherapy (yes vs. no).

Legend: AI, aromatase inhibitor, CMF, cyclophosphamide, methotrexate, and fluorouracil; IEO, European Institute of Oncology; IORT, intraoperative radiotherapy; IQR, interquartile range; n, number; NA, not available; YWS, Young Women's Breast Cancer Study

Supplementary Table 2. Propensity score distribution and covariate balance in patients with node-positive, hormone receptor-positive early breast cancer who completed 5 years of LHRH agonist-based adjuvant endocrine therapy and were assessed for extended endocrine therapy use.

Propensity Score Information													
**Observations**	**Treated (Extended endocrine therapy)**						**Control (No Extended endocrine therapy)**						**Treated - Control**
	**N**	**Weight**	**Mean**	**Standard Deviation**	**Minimum**	**Maximum**	**N**	**Weight**	**Mean**	**Standard Deviation**	**Minimum**	**Maximum**	**Mean Difference**
**All**	287		0.598	0.1176	0.3471	0.9115	214		0.539	0.1125	0.2955	0.8651	0.058
**Region**	287		0.598	0.1176	0.3471	0.9115	214		0.539	0.1125	0.2955	0.8651	0.058
**Weighted**	287	286.21	0.574	0.116	0.3471	0.9115	214	215.35	0.576	0.1241	0.2955	0.8651	-0.001

Legend: N, number.

Supplementary Figure 1. Adjusted Kaplan–Meier curve of Invasive Breast Cancer-Free Survival by receipt of extended endocrine therapy after propensity score weighted analysis and excluding 87 patients with study baseline after 2019.

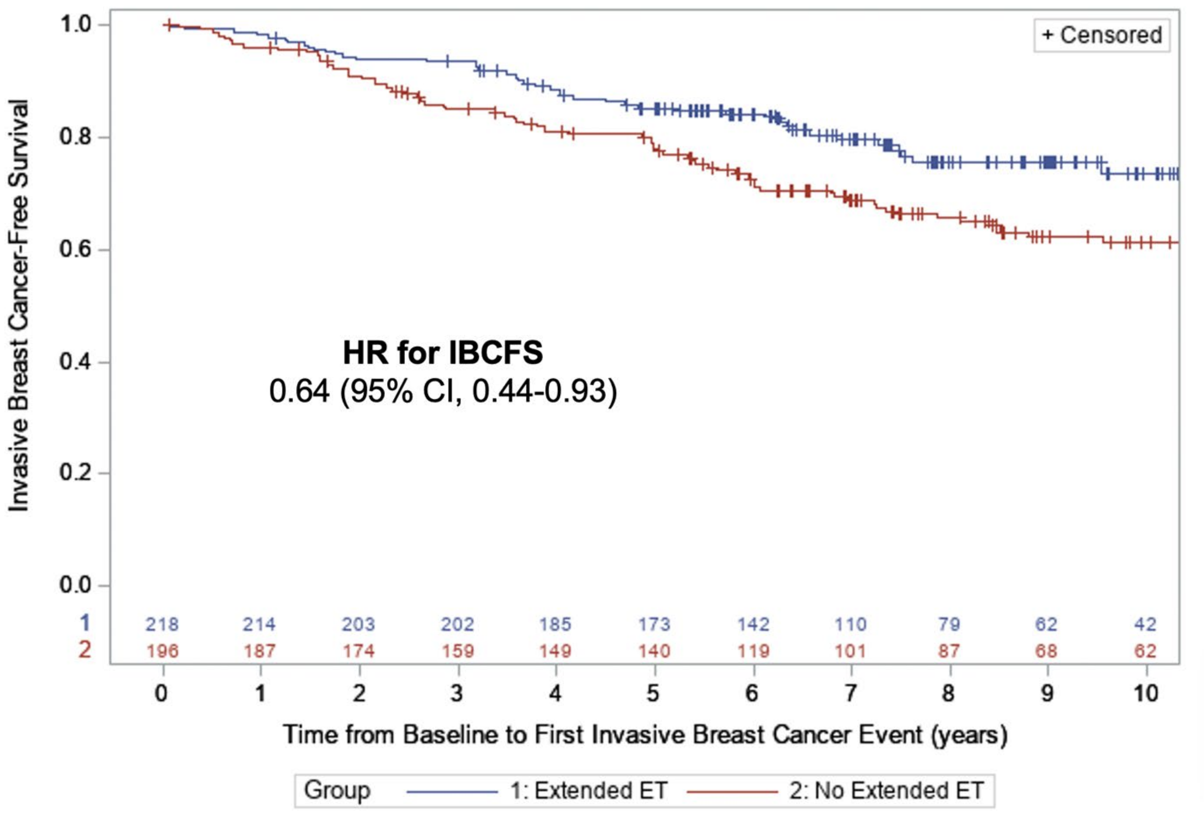

Legend: CI, Confidence Interval; ET, endocrine therapy; HR, Hazard Ratio; IBCFS, Invasive Breast Cancer-Free Survival.
